# The possible relation between temporal muscle mass and glioblastoma multiforme prognosis via sarcopenia perspective

**DOI:** 10.55730/1300-0144.5599

**Published:** 2022-11-20

**Authors:** Osman SÜTCÜOĞLU, Zeynep Sezgi ERDAL, Orhun AKDOĞAN, Emrah ÇELTİKÇİ, Nuriye ÖZDEMİR, Ahmet ÖZET, Murat UÇAR, Ozan YAZICI

**Affiliations:** 1 Department of Medical Oncology, Faculty of Medicine, Gazi University, Ankara, Turkey; 2 Department of Radiology, Faculty of Medicine, Gazi University, Ankara, Turkey; 3 Department of Internal Medicine, Faculty of Medicine, Gazi University, Ankara, Turkey; 4 Department of Neurosurgery, Faculty of Medicine, Gazi University, Ankara, Turkey

**Keywords:** Glioblastome multiforme, temporal muscle, sarcopenia, prognosis

## Abstract

**Background and aim:**

The optimal sarcopenia measurement method in patients with a diagnosis of glioblastoma multiforme (GBM) is unknown. It has been found that temporal muscle thickness (TMT) may reflect sarcopenia and be associated with survival, but the relationship between temporal muscle area (TMA) and GBM prognosis has never been evaluated before. The primary outcome of the study was to evaluate the relationship between TMA/TMT and overall survival (OS) time in newly diagnosed GBM patients.

**Materials and methods:**

The data of patients who presented at the university hospital between January 2009 and January 2019 with a confirmed diagnosis of glioblastoma multiforme at the time of diagnosis were analyzed retrospectively. Temporal muscle thickness and TMA were measured retrospectively from preoperative MRIs of patients diagnosed with GBM. Due to the small number of patients and the failure to determine a cut-off value with acceptable sensitivity and specificity using ROC analysis, the median values were chosen as the cut-off value. The patients were basically divided into two according to their median TMT (6.6 mm) or TMA (452 mm^2^) values, and survival analysis was performed with the Kaplan–Meier analysis.

**Results:**

The median TMT value was 6.6 mm, and the median TMA value was 452 mm^2^. The median overall survival (OS) was calculated as 25.8 months in patients with TMT < 6.6 mm, and 15.8 months in patients with TMT ≥ 6.6 mm (p = 0.29). The median overall survival (OS) of patients with TMA < 452mm^2^ was 26.3 months, and the group with TMA ≥ 452mm^2^ was 14.6 months (p = 0.06). The median disease-free survival was 18.3 months (%95 CI: 13.2–23.4) in patients with TMT < 6.6mm, while mDFS was 10.9 (%95 CI: 8.0–13.8) months in patients with TMT ≥ 6.6mm (p = 0.21). The median disease-free survival was found to be 21.0 months (%95 CI: 15.8–26.1) in patients with TMA < 452 mm^2^ and 10.5 months (%95 CI: 7.8–13.2) in patients with TMA ≥ 452 mm^2^ (p = 0.018).

**Conclusion:**

No association could be demonstrated between TMT or TMA and OS of GBM patients. In addition, the median DFS was found to be longer in patients with low TMA. There is an unmet need to determine the optimal method of sarcopenia in GBM patients.

## 1. Introduction

Glioblastoma multiforme (GBM) is the most common primary malignancy of the brain parenchyma and the most aggressive tumor subtype [1, 2]. The adjuvant treatment of the disease has not changed since the study published by Stupp et al. in 2005, and all patients receive temozolomide concurrent radiotherapy followed by temozolomide chemotherapy [3]. Although all patients undergo similar surgeries and receive the same adjuvant treatments, the disease course is different, and very different disease-free survival (DFS)/overall survival (OS) times are encountered. Therefore, there is a need for a prognostic marker to predict the course of GBM. Age, performance status, type of surgery, and molecular characteristics are previously defined prognostic factors for GBM patients [4]. In addition, it has been shown that performance status is associated with sarcopenia in almost all cancer types, and sarcopenia has been shown to adversely affect cancer prognosis [5–7]. However, the relationship between newly diagnosed GBM and sarcopenia has not been clearly demonstrated.

Measurement of skeletal muscle area at the third lumber (L3) vertebra level is accepted as the gold standard diagnostic method in the diagnosis of sarcopenia [8, 9]. Although the measurement of muscle area at the level of the L3 vertebra is standard, it has been reported that sarcopenia can be diagnosed according to the skeletal muscle area at the level of third cervical (C3) vertebra [10]. Since GBM almost never metastasizes outside of the brain parenchyma, there are no chest or abdominal tomography sections in the imaging of GBM patients, so a clear diagnostic criterion for the evaluation of sarcopenia could not be determined. In addition, limited studies are suggesting that temporal muscle thickness (TMT) correlates with the L3 vertebral skeletal muscle area and may predict sarcopenia [11, 12]. After these studies demonstrating that the temporal muscle may be compatible with sarcopenia, a limited number of studies examining the relationship between TMT and GBM have been reported, but these studies included a relatively small number of patients and the results were inconsistent [13–15]. The gold standard method for sarcopenia is the measurement of the psoas muscle area at the L3 vertebra, and in the validation study, TMT was also found to be correlated with the psoas muscle area [12]. However, the relationship between TMA and psoas muscle area has not been evaluated.

In this study, we aimed to evaluate the relationship of TMA and TMT with overall survival in newly diagnosed GBM patients. The secondary endpoint of the study was the evaluation of the relationship of TMA and TMT with patient and disease characteristics.

## 2. Materials and methods

### 2.1. Study design and patient selection

After the approval of the Gazi University Faculty of Medicine Ethics Committee, the data of the patients who applied to the medical oncology department between January 2009 and January 2019 and whose diagnosis of glioblastoma multiforme was confirmed at the time of diagnosis were retrospectively analyzed. Data collection and data analysis were performed in accordance with ethical standards and the Declaration of Helsinki.

Inclusion criteria were to be older than 18 years of age, have a pathologically confirmed diagnosis of GBM, have completed postoperative chemoradiotherapy, and have received at least 1 course of adjuvant temozolomide. In addition, patients were enrolled who had brain magnetic resonance imaging (MRI) in our center within 30 days before surgery. Patients with pathologies other than GBM Grade IV were not included in the study. Patients with insufficient file data or lost to follow up were excluded from the study. The patients with secondary malignancies were also excluded from study population.

Demographic data such as age at diagnosis, Eastern Cooperative Oncology Group (ECOG) performance status, sex, and tumor size, tumor location, and molecular characteristics of the tumor were recorded. The data of all treatments received by the patients in the postoperative period were scanned. Preoperative complete blood count and serum biochemistry values of the patients were analyzed and neutrophil/lymphocyte ratio (NLR), platelet/lymphocyte ratio (PLR), and systemic inflammation index (SII) values were calculated. Systemic immune-inflammation index (SII) is calculated by (neutrophil × platelet) / lymphocyte. Overall survival (OS) was determined as the time from operation to death. Disease-free survival (DFS) was determined as the time from operation to recurrence of the disease or death. Temporal muscle thickness (TMA) and TMT were calculated by magnetic resonance imaging (MRI) expert radiologists (Z.S.E and M.U.) who were blinded to the clinical features of the patients.

### 2.2. Temporal muscle thickness and area measurement

The brain imaging of all patients was performed with a 3 Tesla MRI scanner (Siemens Magnetom Verio Syngo MR B17, Erlangen, Germany) using 12-channel head coil. Axial T1 (repetition time [TR]: 150–160 ms, echo time [TE]: 2 ms, slice thickness: 1 mm; field of view (FOV): 210 mm) weighted image, oriented parallel to the anterior commissure-posterior commissure line were used for measurements.

**Figure 1:**
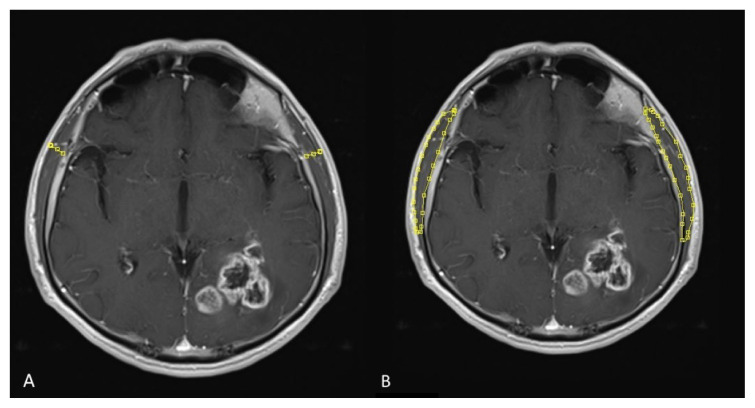
Temporal muscle thickness (TMT) (A) and temporal muscle area (TMA) (B) measurements on contrast-enhanced axial plain T1 weighted images.

MRI images were evaluated by the same radiologist blinded to the clinical information of the patients. Temporal muscle thickness and TMA was measured as perpendicular to the largest axis on transverse section. The surface area of temporal muscle (TMA) was calculated in semiquantitative volumetric method as the largest surface area from the section (Figures 1A and 1B). The orbital roof and the Sylvian fissure on T1-weighted images were used as anatomical landmarks. The TMA for both right and left temporal muscles were given in mm^2^. Each patient’s mean TMT and TMA were calculated by measuring the left and right sides independently, adding them up, and dividing by half. If there were any indications of prior intervention on one side that could have affected temporal muscle thickness or area (such as prior craniotomy, muscle edema, or subsequent muscle atrophy), this side was excluded from measurements, and only the temporal muscle of this patient’s other side was used for further analysis.

### 2.3. Statistical analysis

Statistical analyses were performed using SPSS version 23 (IBM Corp., Armonk, NY, USA). To evaluate the normal distribution of continuous variables, visual (histogram and probability charts) and analytical methods (the Kolmogorov–Smirnov/Shapiro–Wilk tests) were used. For nonnormally distributed variables, descriptive analyses were presented as median ± min/max values. Categorical data are expressed as numbers and percentages (%). The chi-square or Fisher’s exact test was used to evaluate the relationship between TMT and TMA and categorical variables. BMI was divided into 3 categorical groups as <25, 25–30, and >30. Pearson and chi-square tests were used to evaluate the relationship of BMI groups with TMT and TMA. Distribution analyses were performed for TMT, TMA, NLR, PLR, and SII values, and cut-off values were determined according to their medians. Since a cut-off value with sufficient sensitivity and specificity could not be found with ROC analysis, groups were formed according to median values. The patients were divided into two groups according to these cut-off values. We used 2 different models for survival analysis. For univariate analysis, the Kaplan–Meier analysis and log-rank analysis was performed, and hazard ratio (HR) was calculated using Cox proportional hazard regression models. Possible factors determined by univariate analyses were evaluated by Cox regression analysis with backward selection to determine independent predictors of the overall survival rate of GBM. HR values determined by multivariate analysis are presented with a 95% confidence interval (95% CI). In the interpretation of all analyses, the p < 0.05 value was considered statistically significant.

## 3. Results

A total of 74 patients who met the inclusion criteria were included in the study. The majority of the patients were male (n = 46, 62%) and the median age of the patients was 57 (min–max: 25–76). Gross total resection of the tumor was performed in 70% of the patients. The characteristic data of the patients are presented in Table 1.

In all patients, the median TMT value was 6.6 mm, and the median TMA value was 452 mm^2^. Median values for NLR, PLR, and SII were calculated and found to be 5.97, 159.6, and 1366, respectively. TMT and TMA values of the patients according to age, sex, ECOG performance status, tumor location, and tumor size are presented in Table 2. Temporal muscle thickness was found to be thicker in patients younger than 65 years of age and patients with tumors located in the right hemisphere (respectively, p = 0.048 and p = 0.006). In the analysis of the relationship between TMA and subgroups, TMA was found to be greater in patients younger than 65 years of age (p = 0.025) in the male sex (p = 0.006), and patients with a right-located tumor (p = 0.033). There was no significant difference for TMT and TMA parameters among BMI grouping as BMI < 25, BMI = 25–30 and BMI > 30 kg/m^2^ (p = 0.123 and p = 0.996).

**Figure 2:**
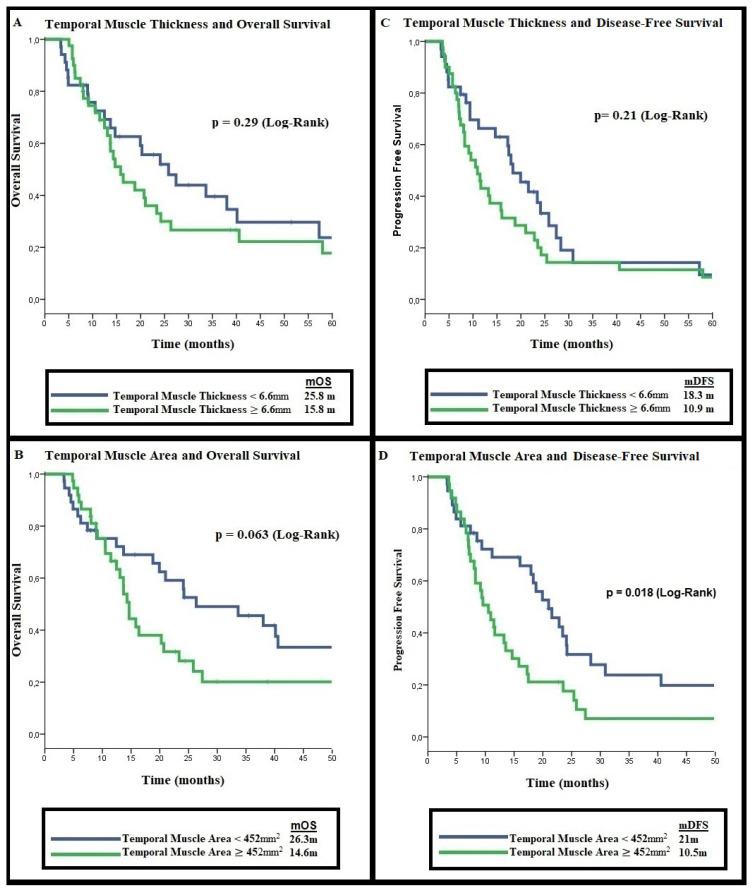
Temporal muscle measurements and overall/progression-free survival. A: Temporal muscle thickness and overall survival, B: Temporal muscle thickness and progression-free survival, C: Temporal muscle area and overall survival, D: Temporal muscle area and progression-free survival.

The median overall survival (mOS) of the patients included in the study was calculated as 20.2 months (%95 CI: 12.1–28.3). Since our patients have been diagnosed since 2009, most of them did not have molecular analysis. The isocitrate dehydrogenase (IDH) mutation status of 32 (43%) of the patients was known (27 IDH wild, 5 IDH mutant). The mOS of IDH mutant patients was numerically shorter than the IDH wild group (12.2 months vs 26.3 months, p = 0.52). There was no significant difference between the mDFS of IDH mutant patients and the IDH wild group (9.3 months vs. 11.6 months, p = 0.911). Being younger than 65 years and having an ECOG performance score < 2 were associated with longer mOS (p = 0.001 and p = 0.003, respectively). The median overall survival was calculated as 25.8 months (%95 CI: 14.8–36.8) in patients with TMT < 6.6 mm, and 15.8 months (%95 CI: 12.1–19.5) in patients with TMT ≥ 6.6 mm (p = 0.29). The median overall survival of patients with TMA < 452 mm^2^ was 26.3 months (%95 CI: 9.2–43.4) and the group with TMA ≥ 452 mm^2^ was 14.6 months (%95 CI: 13.3–16.0) (p = 0.06) (Figures 2A and 2B). Univariate and multivariate analyses with factors affecting survival are presented in Table 3.

**Figure 3:**
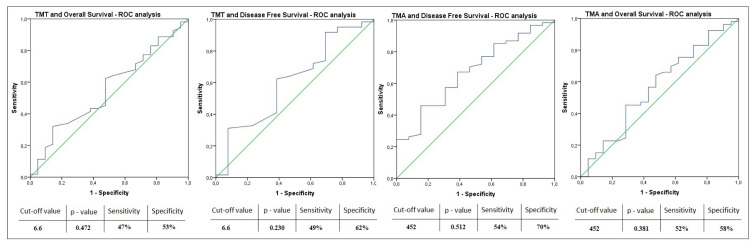
ROC analyses of the relationship between TMT and TMA median values and DFS and OS.

The median disease-free survival (mDFS) of the patients was calculated as 14.6 months (%95 CI: 8.3–20.9). The median disease-free survival was 18.3 months (%95 CI: 13.2–23.4) in patients with TMT < 6.6 mm, while mDFS was 10.9 (%95 CI: 8.0–13.8) months in patients with TMT ≥ 6.6 mm (p = 0.21). The median disease-free survival (DFS) analysis according to the TMA, the mDFS was found to be 21.0 months (%95 CI: 15.8–26.1) in patients with TMA < 452 mm^2^ and 10.5 months (%95 CI: 7.8–13.2) in patients with TMA ≥ 452 mm^2^ (p = 0.018) (Figures 2C and 2D). In the evaluation of the relationship between DFS and other factors, mDFS was found to be longer in the group with low PLR (p = 0.03). In multivariate analysis, both PLR (HR: 2.1, %95 CI: 1.15–3.85, p = 0.015) and TMA (HR:1.87, %95 CI: 1.03–3.38, p = 0.036) were found to be associated with mDFS. The results of univariate and multivariate analyses evaluating the factors affecting the prognosis of disease-free survival are presented in Table 4. ROC analyzes of the relationship between TMT and TMA median values and DFS and OS are presented in Figure 3.

## 4. Discussion

Numerous studies have shown that sarcopenia affects cancer prognosis, but the optimal sarcopenia diagnosis method in GBM patients has not been defined yet [12, 16, 17]. Since classical sarcopenia measurement methods are impractical in GBM patients, TMT was thought to be predictive for the diagnosis of sarcopenia. In our study, there was no relation between TMT and mOS or mDFS; however, there was a relation between TMA and mDFS. To the best of our knowledge, this is the first study to evaluate the relationship between TMA and survival in newly diagnosed GBM patients.

Although the factors affecting TMT are not clearly known, the relationship between some factors and TMT has been shown. In a study conducted in patients with lung and breast cancer, TMT was found to be thicker in younger patients and male sex [18, 19]. On the other hand, in a study conducted in patients with GBM, it was determined that BMI directly affects TMT [20]. It has also been shown that ECOG performance status and drugs (especially corticosteroids) can affect TMT [14]. Since the patients in the studies were all of the same race, the effect of the patients’ ethnicity on the TMT could not be clearly demonstrated. Similar to the literature, we identified an inversely proportionate relationship between age and TMT in our study. Another important issue is to determine the factors affecting TMA. A relationship was found between TMA and age, sex and tumor hemisphere. Additional studies are needed to determine optimal cutoff values for TMA and TMT based on race, age, and perhaps BMI.

A limited number of studies in the literature have shown the relationship between TMT and GBM prognosis [21, 22]. In a metaanalysis published in 2021, studies in newly diagnosed GBM patients were evaluated, and it was reported that low TMT was associated with poor GBM prognosis [23]. In addition, although an optimal cut-off for TMT has not been determined, sex-specific cut-off values have been tried to be standardized in a recently published study [24]. However, as we know from sarcopenia studies, cut-off values in muscle area/muscle thickness measurements may differ according to race, age, and sex [24]. In addition, although there is no study examining the relationship between TMT and sarcopenia in patients with GBM, inferences were made from the results of studies in other diseases [25, 26]. Contrary to the literature, in our study, no relationship was found between overall survival or progression-free survival and TMT. Even if it is not statistically significant, the group with TMT below the median has numerically longer OS and DFS. There may be many factors that can create this different result; one of these reasons may be the different determination of TMT cut-off value in almost all studies. Other reasons for the contrasting results between studies may be the small number of patients in the studies, different characteristics of tumors (tumor size, multifocal tumor, subtotal/gross total resection), and molecular characteristics of the tumor. In particular, with the updated glial tumor classification of the World Health Organization in 2021, IDH mutant tumors were not classified as GBM and were evaluated as astrocytomas [27]. Since the IDH mutation and MGMT (O6-methylguanine-DNA-methyltransferase) methylation status of the patients in our study were not known, perhaps the different results are directly affected by the molecular characteristics of the tumors.

Measurement of muscle area at the level of the L3 vertebra is used as the gold standard diagnostic method for sarcopenia [28–31]. This method has some difficulties; the main difficulty is that it takes a long time, and every patient needs to have an abdominal tomography or MRI. Again, in this measurement, the height of the patients should be known, and this may create difficulties for retrospective studies. TMT and TMA measurements can be done quite easily, and it has been reported that a patient’s TMT measurement takes less than 1 min [32]. Our study may indicate that TMA may be more sensitive than TMT because while no relationship could be shown between TMT and both OS and DFS, it was determined that TMA could affect the DFS. On the other hand, it is known that there is a strong correlation between mOS and mDFS in GBM patients. However, in our study, although a statistically significant relationship was found between TMA and mDFS, the relationship between TMA and mOS could not be proven. Furthermore, the median overall survival time was 11.7 months longer in the patient group with TMA < 452 mm^2^. In fact, our results seem to have a similar trend for PFS and OS. The fact that this correlation was not shown statistically can be explained by the low number of patients and the unknown molecular properties. Additional studies are needed to evaluate the relationship between TMA and sarcopenia and to investigate TMA and cancer prognosis.

The relationship between indices that can be calculated using complete blood count parameters and cancer prognosis has been evaluated many times. Since neutrophils, platelets, and lymphocytes are involved in inflammatory processes, NLR, PLR, and SII indices are considered to be important indicators of systemic inflammation [27, 28]. Increased inflammatory cytokines have been shown to induce muscle wasting, inhibit muscle synthesis and increase protein catabolism [27]. Therefore, increases in these indices are found to be associated with shorter survival times in many cancers [29, 32]. On the other hand, the relationship between these indices and GBM could not be clearly demonstrated, and contradictory results were found. Although the independent effect of increased PLR values on GBM survival was demonstrated in the study by Wang et al., this relationship could not be demonstrated in the other three studies analyzing this issue [33–36]. In our study, it was concluded that there was no relationship between PLR and overall survival, but increased PLR value was found to be associated with shorter DFS. These different outcomes may be related to different stages of patients (new diagnosis vs. relapse), different molecular characteristics (IDH wild/mutant/unknown), surgical quality, and tumor characteristics. For this reason, it can be difficult to predict survival based on markers that reflect the patient’s immune system alone. It would be a correct approach to evaluate the relationship between these indices and survival in a larger patient group, where tumor characteristics and molecular features are similar and patient-related factors are comparable.

Our study has certain limitations. Having a retrospective design and insufficient data on tumor molecular characteristics are the biggest limitations in the interpretation of the results. On the other hand, there is no internationally accepted cut-off value for TMT and TMA. Therefore, the optimal cut-off value could not be determined, and the patients were divided into two groups according to the median values. In addition, in context of the sarcopenia-inducing effect of steroids, our lack of information regarding the patients’ steroid use patterns can be considered a limitation. Another limitation is that most of the patients (74%) are younger than 65 years of age. Sarcopenia is known to be age-related; and thus, results may vary in a larger study of patients older than 65 years. Besides these limitations, when the data of our study were analyzed, it was determined that the DFS and OS times were relatively high. In this case, all patients were patients who had undergone surgery, completed adjuvant chemoradiotherapy, and completed at least 1 course of temozolomide therapy so that the patients had similar clinical characteristics. Therefore, we think that we selected a relatively good patient group and that the OS and DFS times were relatively high. However, the unknown molecular pathological features of the tumor, such as IDH mutation and MGMT methylation, preclude the homogeneous distribution in our study. In this context, we are planning a new clinical trial with a larger number of patients with similar molecular characteristics and clinical features.

In conclusion, no association could be demonstrated between TMT or TMA and OS of GBM patients; however, there is a strong association between TMA and DFS. There is an unmet need to determine the optimal method of sarcopenia in GBM patients. Studies on the correlation of TMA with gold standard sarcopenia measurements are needed, and these studies, especially those involving elderly patients, may contribute to a new sarcopenia marker literature.

## Figures and Tables

**Table 1:** Characteristics of the study population.

Group		Number	(%)
Age			
	<65 years	55	(74%)
	≥65 years	19	(26%)
Sex			
	Female	28	(38%)
	Male	46	(62%)
ECOG performance status			
	0–1	59	(80%)
	≥2	15	(29%)
Operation			
	Subtotal excision	52	(70%)
	Gross total excision	22	(30%)
Tumor diameter			
	<30 mm	38	(51%)
	≥30mm	36	(49%)
Tumor hemisphere			
	Right	34	(46%)
	Left	40	(54%)
Body mass index status (kg/m^2^)			
	<25	25	(34%)
	25–30	37	(50%)
	>30	12	(16%)
		**Median**	**(min–max)**
Temporal muscle thickness (mm)		6.6	(2.8–16.8)
Temporal muscle area (mm^2^)		452	(154–803)
Hemoglobin (g/dL)		13.6	(9.0–16.5)
Platelet (/μL)		243,000	(85000–476000)
Absolute neutrophil count (/μL)		7900	(1700–27000)
Absolute lymphocyte count (/μL)		1300	(470–6200)

ECOG: Eastern Cooperative Oncology Group

**Table 2:** Temporal muscle thickness and area measurement according to patients’ characteristics.

Group		TMT median (min–max)	p–value	TMA median (min–max)	p-value
Age					
	<65 years	6.7 (3.1–16.8)	**0.048**	461 (161–803)	**0.025**
	≥65 years	5.9 (2.8–12.2)		378 (154–627)	
Sex					
	Female	6.7 (2.8–16.8)	0.631	396 (154–776)	**0.006**
	Male	6.6 (4.4–12.2)		492 (213–803)	
ECOG performance status					
	0–1	6.6 (2.8–16.8)	0.657	459 (154–803)	0.586
	≥2	6.8 (3.1–10.3)		399 (161–657)	
Operation					
	Subtotal excision	6.7 (3.1–16.8)	0.232	455 (161–803)	0.178
	Gross total excision	6.4 (2.8–10.0)		414 (154–681)	
Tumor diameter					
	<30 mm	6.4 (3.1–12.2)	0.205	380 (160–600)	0.006
	≥30mm	6.8 (2.8–16.8)		493 (154–803)	
Tumor hemisphere					
	Right	7.4 (4.7–16.8)	**0.006**	468 (213–803)	**0.033**
	Left	6.4 (2.8–10.3)		403 (154–627)	
Body mass index status (kg/m^2^) (kg/m^2^)					
	<25	6.3 (4.6–10.3)	0.123	462 (213–603)	0.996
	25–30	6.8 (4.4–9.4)		468 (187–803)	
	>30	7.4 (2.8–16.8)		468 (154–776)	

**ECOG:** Eastern Cooperative Oncology Group,

**TMT:** Temporal Muscle Thickness,

**TMA:** Temporal Muscle Area.

**Table 3:** Univariate and multivariate analyses of factors for the prognosis of overall survival.

Characteristics		n, %	mOS (months)	Univariate analyses HR (95% CI) p-value		Multivariate analyses HR (95% CI) p-value	
Age							
	<65 years	55 (62%)	23.3	1.00			
	≥65 years	19 (38%)	9.0	**2.73 (1.48–5.05)**	**0.001**	**2.93 (1.57–5.45)**	**0.001**
Sex							
	Female	28 (30%)	18.8	1.00			
	Male	46 (70%)	20.7	0.74 (0.42–1.31)	0.30	**–**	**-**
ECOG							
	0–1	59 (67%)	23.3	1.00			
	≥2	15 (23%)	8.0	**2.63 (1.36–5.09)**	**0.003**	**2.87 (1.46–5.64)**	**0.002**
Hemisphere location							
	Right	34 (46%)	18.8	1.00			
	Left	40 (54%)	20.2	0.85 (0.49 –1.47)	0.51	**-**	**-**
Tumor diameter							
	<30 mm	38 (51%)	20.2	1.00			
	≥30 mm	36 (49%)	15.8	1.09 (0.63–1.88)	0.73	**-**	**-**
TMT							
	<6.6 mm	34 (43%)	25.8	1.00			
	≥6.6 mm	40 (57%)	15.8	1.34 (0.77–2.32)	0.29	**-**	**-**
TMA							
	< 452 mm^2^	37 (50%)	26.3	1.00			
	≥ 452 mm^2^	37 (50%)	14.6	1.69 (0.96–2.96)	0.06	**-**	**-**
NLR							
	Low NLR	37 (50%)	18.8	1.00			
	High NLR	37 (50%)	14.6	1.67 (1.11–2.51)	0.63	-	-
PLR							
	Low PLR	37 (50%)	16.3	1.00			
	High PLR	37 (50%)	14.6	1.33 (0.72–2.48)	0.35	-	-
SII							
	Low SII	37 (50%)	18.8	1.00			
	High SII	37 (50%)	14.6	1.12 (0.60–2.08)	0.71	-	**-**
AGR							
	Low AGR	37 (50%)	14.2	1.00			
	High AGR	37 (50%)	18.1	0.83 (0.44–1.55)	0.56	**-**	**-**

**ECOG:** Eastern Cooperative Oncology Group,

**TMT:** Temporal Muscle Thickness,

**TMA:** Temporal Muscle Area.

**Table 4:** Univariate and multivariate analyses of factors for the prognosis of disease-free survival.

Characteristics		n, %	mOS (months)	Univariate analyses HR (95% CI) p-value		Multivariate analyses HR (95% CI) p-value	
Age							
	<65 years	55 (62%)	17.2	1.00			
	≥65 years	19 (38%)	8.3	1.81 (0.99–3.30)	0.05	**-**	
Sex							
	Female	28 (30%)	15.8	1.00			
	Male	46 (70%)	13.3	1.02 (0.60–1.74)	0.93	**-**	**-**
ECOG							
	0–1	59 (67%)	17.7	1.00			
	≥2	15 (23%)	7.4	1.82 (0.96–3.47)	0.06	**-**	**-**
Hemisphere location							
	Right	40 (59%)	13.5	1.00			
	Left	34 (31%)	16.0	0.93 (0.56 –1.54)	0.78	**-**	**-**
Tumor diameter							
	<30 mm	38 (72%)	17.1	1.00			
	≥30 mm	36 (28%)	11.6	1.22 (0.73–2.02)	0.43	**-**	**-**
TMT							
	<6.6 mm	34 (43%)	18.3	1.00			
	≥6.6 mm	40 (57%)	10.9	1.37 (0.82–2.29)	0.21	**-**	**-**
TMA							
	< 452 mm^2^	37 (50%)	21.0	1.00			
	≥ 452 mm^2^	37 (50%)	10.5	**1.84 (1.10–3.08)**	**0.018**	**1.87 (1.03–3.38)**	**0.036**
NLR							
	Low NLR	37 (50%)	11.6	1.00			
	High NLR	37 (50%)	11.4	0.63 (0.35–1.15)	0.13	-	-
PLR							
	Low PLR	37 (50%)	14.6	1.00			
	High PLR	37 (50%)	10.5	**1.94 (1.05–3.43)**	**0.03**	**2.1 (1.15–3.85)**	**0.015**
SII							
	Low SII	37 (50%)	11.4	1.00			
	High SII	37 (50%)	11.6	0.77 (0.43–1.39)	0.39	-	**-**
AGR							
	Low AGR	37 (50%)	11.1	1.00			
	High AGR	37 (50%)	11.6	0.88 (0.50–1.55)	0.66	**-**	**-**

**ECOG:** Eastern Cooperative Oncology Group,

**TMT:** Temporal Muscle Thickness,

**TMA:** Temporal Muscle Area,

**NLR**: Neutrophil Lymphocyte ratio,

**PLR**: Platelet Lymphocyte Ratio,

**SII:** Systemic Inflammation Index,

AGR: Albumin Globulin Ratio.
